# Pulsed field ablation-based pulmonary vein isolation in atrial fibrillation patients with cardiac implantable electronic devices: practical approach and device interrogation (PFA in CIEDs)

**DOI:** 10.1007/s10840-022-01445-0

**Published:** 2022-12-08

**Authors:** Shaojie Chen, Julian K. R. Chun, Stefano Bordignon, Shota Tohoku, Lukas Urbanek, David Schaack, Ramin Ebrahimi, Britta Schulte-Hahn, Boris Schmidt

**Affiliations:** 1 Cardioangiologisches Centrum Bethanien (CCB), Frankfurt Academy For Arrhythmias (FAFA), Kardiologie, Medizinische Klinik III, Agaplesion Markus Krankenhaus, Akademisches Lehrkrankenhaus der Goethe-Universität Frankfurt am Main, Frankfurt Am Main, Germany; 2 Die Sektion Medizin, Universität Zu Lübeck, Lübeck, Germany

## Introduction

Catheter ablation is an effective rhythm control strategy in treating atrial fibrillation (AF) [1–5].

Pulmonary vein isolation (PVI) remains the cornerstone of AF ablation [1–7].

Among the aging population, because of a wide spectrum of conditions that could require device implantation and the rising prevalence of AF, there has been increasing number of patients with cardiac implantable electrical devices (CIEDs) who also suffer from AF.

Pulsed field ablation (PFA) is emerging as novel non-thermal ablation technology. PFA has gained great interest given its notable safety and efficacy profile, e.g., myocardial tissue selectivity and unique ability to reduce the risk of collateral tissue damage [8–10]. Initial clinical data have showed that PFA represents a powerful ablation technology and allows for fast ablation [11–15].

However, (1) PFA in patients with CIEDs has not been systematically reported and (2) potential PFA interactions with CIEDs remain unclear. In this study, we report the procedural approach, feasibility, and safety of PFA for AF in patients with CIEDs.

## Methods

### Study population

PFA has been performed at the Cardioangiologisches Centrum Bethanien (CCB) of Markus Hospital, Frankfurt am Main, Germany. Patients with symptomatic paroxysmal or persistent AF underwent the index PFA-based PVI. In this study, patients with CIEDs including pacemaker, implantable cardioverter-defibrillators (ICD), or cardiac resynchronization therapy plus defibrillator (CRT-D) were consecutively included. Baseline characteristics and procedural data were collected. The data analysis complied with the Declaration of Helsinki and was reviewed by the institutional board. All patients provided written informed consent before the procedures.

### Procedure

Four experienced electrophysiologists (S. C., J. C., B. S., S. B.) performed the procedures as the primary operator using the same institutional approach. Conventionally, no pre-procedural cardiac computed tomography or magnetic resonance imaging was required. Transesophageal echocardiography (TEE) was performed to evaluate the cardiac anatomy and exclude intracardiac thrombus. If the patients were under vitamin K antagonists (VKA) therapy, the VKA was uninterrupted. If the patients were under non-vitamin K antagonist oral anticoagulants (NOAC) therapy, the morning dose was paused and continued 6 h after the procedure.

Patients were carefully sedated by intravenously administering of midazolam and propofol. Intravenous unfractionated heparin (100 U/kg) was given targeting activated clotting time (ACT) 300–350 s. After two femoral venous punctures, one multipolar diagnostic catheter (6F, Inquiry; Abbott) was placed in the coronary sinus (CS), and single-transseptal puncture (SL1, 8.5F; Abbott) was performed under fluoroscopic and pressure guidance. Selective pulmonary vein (PV) angiography for was performed in projections of RAO 30° and LAO 40°.

### The PFA system

The PFA system consists of (1) a generator which delivers pulsed electrical waveforms over multiple channels (Farastar, Farapulse Inc., Menlo Park, California), (2) a 13-F steerable delivery sheath (Faradrive), and (3) a PFA ablation catheter (Farawave).

The 12-F PFA ablation catheter (Farawave) contains 5 splines, each containing 4 electrodes to deliver pulsed field ablation energy. The PFA ablation catheter can be progressively configured into different poses: from a baseline linear shape for introducing the PFA catheter into the steerable sheath, to a semi-deployed ball or basket pose, and to a fully deployed flower configuration. Two catheter sizes were available: 31 or 35 mm at full deployment.

### Ablation procedure

The parameters of the CIEDs (baseline and the same day after the ablation procedure), including threshold, sensing amplitude, and impedance of the atrial and/or ventricular leads, were interrogated under sinus rhythm. The anti-tachycardia therapy of the ICDs was deactivated before the ablation and reactivated directly after the procedure. The modes of the devices were kept unchanged throughout the procedure.

The transseptal sheath was then exchanged with the 13-F steerable delivery sheath (Faradrive) using over-the-wire technique into the left atrium (LA). The sheath was continuously flushed with heparinized saline at 20 ml/h.

The PFA ablation catheter (Farawave) was then advanced via the steerable delivery sheath over a guide wire into the LA to achieve the PVs. PFA ablation started at the left superior pulmonary vein (LSPV) and was carried out in a clockwise fashion (LSPV, left inferior pulmonary vein (LIPV), right inferior pulmonary vein (RIPV), and right superior pulmonary vein (RSPV).

The ablation energy was delivered with a set of microsecond scale, biphasic, unsynchronized 1.9–2.0-kV pulses. The duration of each PFA application, consisting of 5 trains of pulses, was 2.5 s.

Baseline PV potentials were recorded from all PVs. No 3D electroanatomic mapping system was used in this cohort. Each PV was ablated with 8 applications using two different configurations guided by fluoroscopy and baseline PV angiograms; the “8 applications protocol” were 2 in basket configuration—> small rotation (for lesion overlapping)—> another 2 in basket configuration—> 2 in flower configuration (for PV antral lesion)—> small rotation—> another 2 in flower configuration. Importantly, any ablations close to the leads or devices were avoided.

Phrenic nerve function was evaluated by direct phrenic capture and by observing diaphragmatic motion during inspiration. Luminal esophageal temperature monitoring was not performed.

Single-shot PVI was defined as the elimination of the PV spike potentials after the first energy application at the respective PV. After ablation, PVs were re-mapped and PVI was confirmed by electrograms with and without differential pacing. After the procedure, the CIEDs were re-interrogated and ICD was reactivated.

### Post-procedure care and follow-up

All patients received transthoracic echocardiography to exclude pericardial effusion. The evening dose of anticoagulation was resumed the after the procedure. A 24-h Holter ECG was obtained before discharge to exclude early arrhythmia recurrence. Antiarrhythmic drugs (AAD) were halted after the procedure. All patients were scheduled for outpatient clinic visits at 3, 6, and 12 months including CIEDs interrogation and transthoracic echocardiography.

### Statistical analysis

Continuous variables were described as mean ± SD, and discrete variables were reported as number and percentage. *P* values < 0.05 (two-tailed) were considered significant. All statistical analyses were performed using the SPSS software (Version 22.0, SPSS Inc.).

## Results

As summarized in Table 1, a total of 20 patients were included, of them 12 (60%) had dual-chamber pacemaker (DDD), 2 (10%) had single-chamber pacemaker (VVI), 3 (15%) had ICD, and 3 (15%) had CRT-D. Mean age was 71.7 ± 12.3 years, and 35% were female. Paroxysmal AF or persistent AF was 65% or 35% respectively. The mean CHA_2_DS_2_-VASc Score was 4.6 ± 1.6, mean left atrium (LA) diameter was 41.6 ± 4.4 mm, and mean left ventricular ejection fraction (LVEF) was 54.0 ± 14.6%.

**Table 1:** Summary of demographic data

*N*	20
Age, years	71.7 ± 12.3
Female gender, %	35%
BMI, kg/m^2^	25.8 ± 3.3
Par-AF/Per-AF, %	65%/35%
Hypertension, %	70%
Diabetes mellitus, %	10%
Previous stroke, %	0%
Heart failure, %	45%
Coronary heart disease, %	30%
CHA_2_DS_2_-VASc Score	4.6 ± 1.6
LA, mm	41.6 ± 4.4
LVEF, %	54.0 ± 14.6
Refractory AADs	1.2 ± 0.4
Dual-chamber pacemaker (DDD)	60%
Single-chamber pacemaker (VVI)	10%
ICD	15%
CRT-D	15%

*AF*, atrial fibrillation; *Par-AF*, paroxysmal atrial fibrillation; *Per-AF*, persistent atrial fibrillation; *LA*, left atrium; *LVEF*, left ventricular ejection fraction; *AAD*, antiarrhythmic drug; *ICD*, implantable cardioverter-defibrillator; *CRT-D*, cardiac resynchronization therapy plus defibrillator

**Fig. 1:**
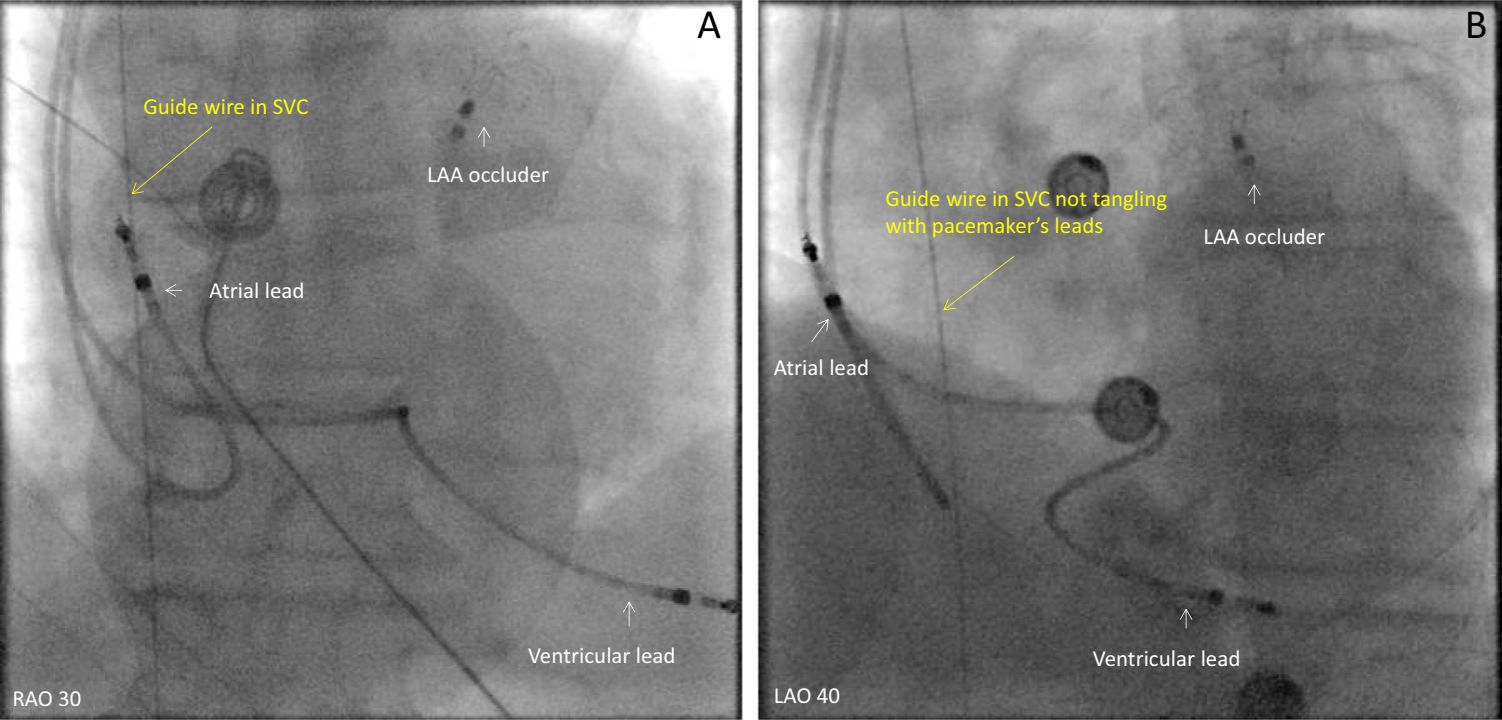
Baseline fluoroscopic position of the cardiac device

**Fig. 2:**
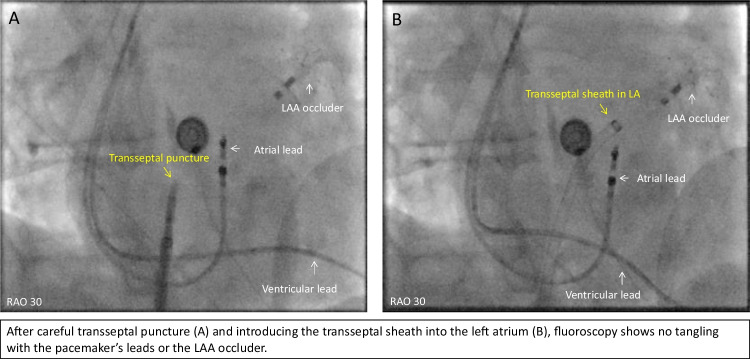
Transseptal puncture under guidance of fluoroscopy

**Fig. 3:**
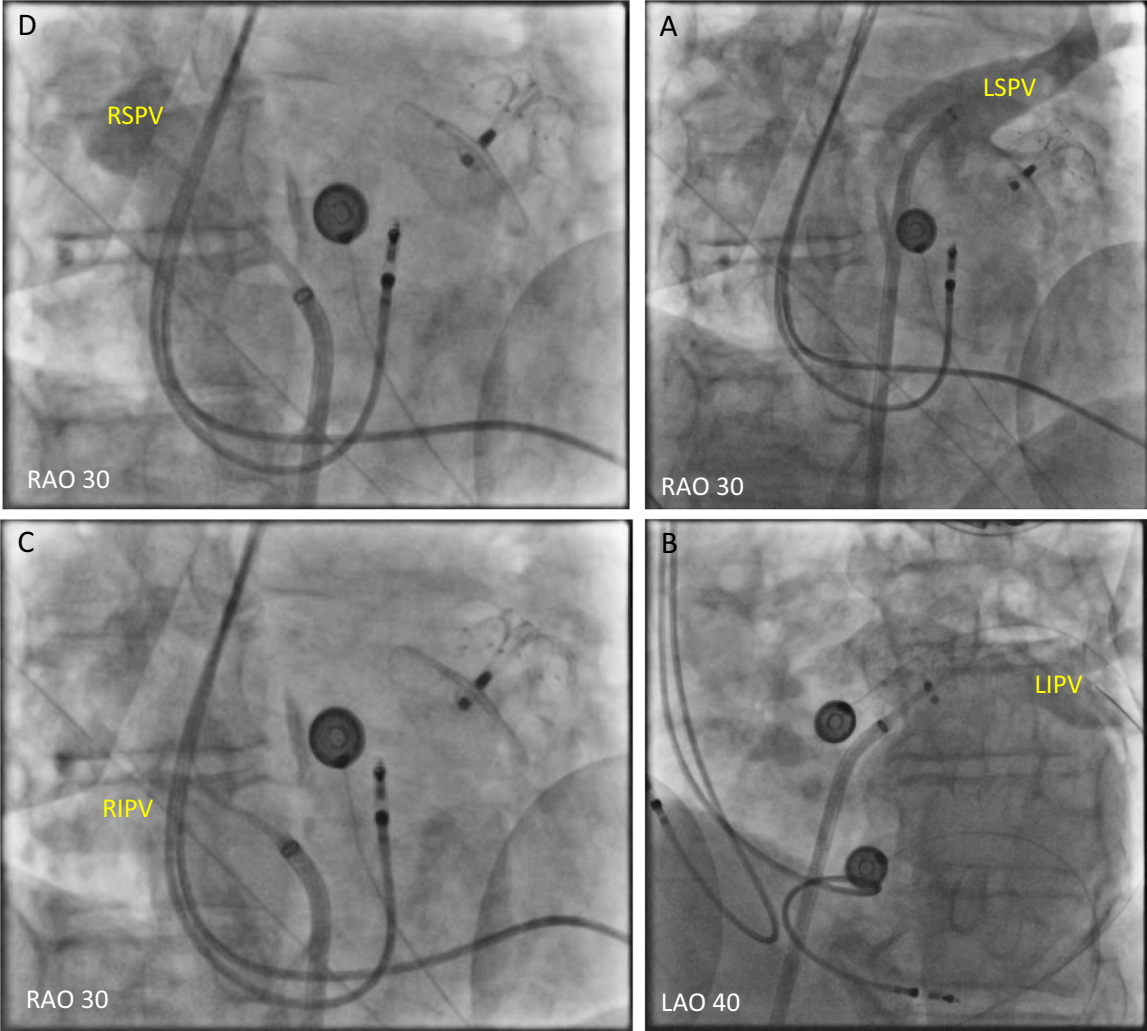
Angiography of pulmonary veins at baseline

**Fig. 4:**
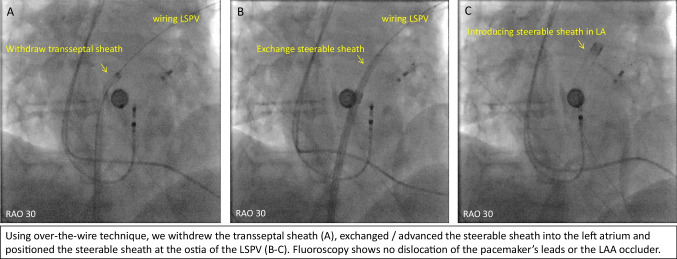
Exchange of steerable sheath into the left atrium using over-the-wire technique

**Fig. 5:**
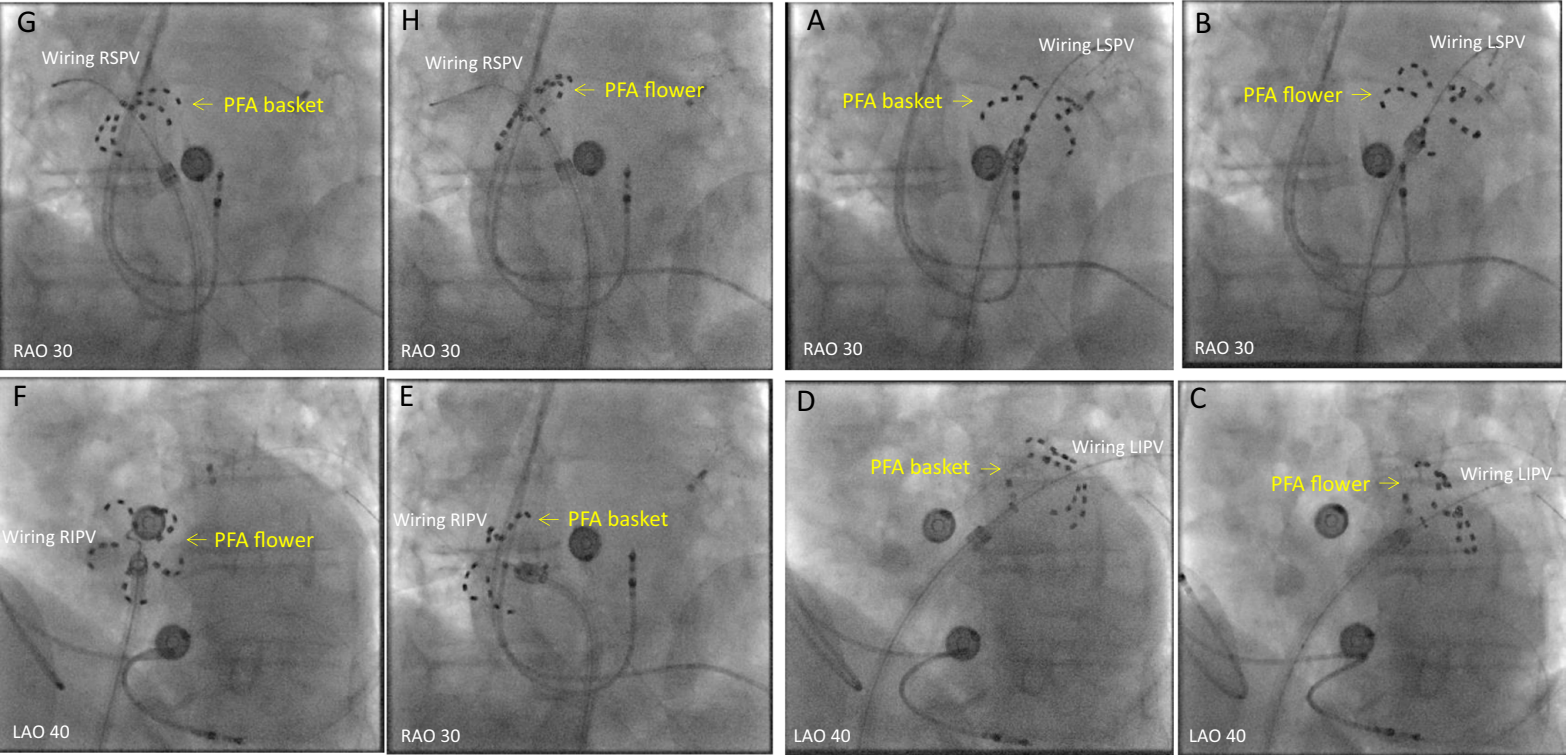
Pulsed field ablation approach of each pulmonary vein

**Fig. 6:**
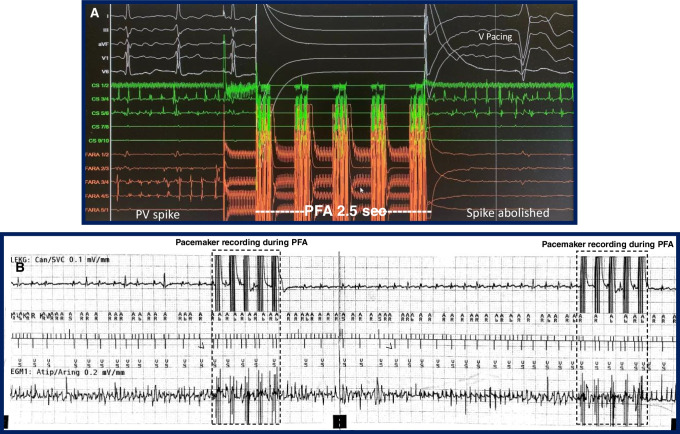
Electro gram and pacemaker recording during pulsed field ablation

Figures 1, 2, 3, 4, and 5 are representative figures step-by- step showing PFA procedure in a patient with a DDD pacemaker and a LAA occluder. Figure 1 shows the baseline fluoroscopic position of the atrial lead and the ventricular lead, and a long guide wire is placed at superior vena cava (SVC), importantly without tangling with the pacemaker’s leads confirmed by different projections of the fluoroscopy. Figure 2 shows the transseptal puncture under the guidance of fluoroscopy. After careful transseptal puncture and introducing the transseptal sheath into the left atrium, fluoroscopy shows no tangling with the pacemaker’s leads or the LAA occluder. Figure 3 shows the baseline angiography of the LSPV, LIPV, RIPV, and RSPV. Figure 4 shows the exchange of a long steerable sheath into the left atrium (LA) using over-the-wire technique, during which fluoroscopy shows no dislocation of the pacemaker’s leads or the LAA occluder. Figure 5 shows PFA of LSPV, LIPV, RIPV, and RSPV using different configurations (Fig. 5A–H). Figure 6A shows the elimination of PV potentials after first PFA application and ventricular pacing directly after the PFA application because of transient bradycardia. Figure 6B shows the pacemaker recording (atrial/ventricular lead sensing during PFA applications).

We observed the real-time electrogram (EGM) during PFA energy delivery from the included 20 patients. Eight out of 20 (40%) patients were pacemaker dependent, who were in DDD mode, the duration of each PFA application was 2.5 s, during which sensing EGMs at the leads (atrial/ventricular) of the pacemaker and no pacemaker inhibition was observed.

**Fig. 7:**
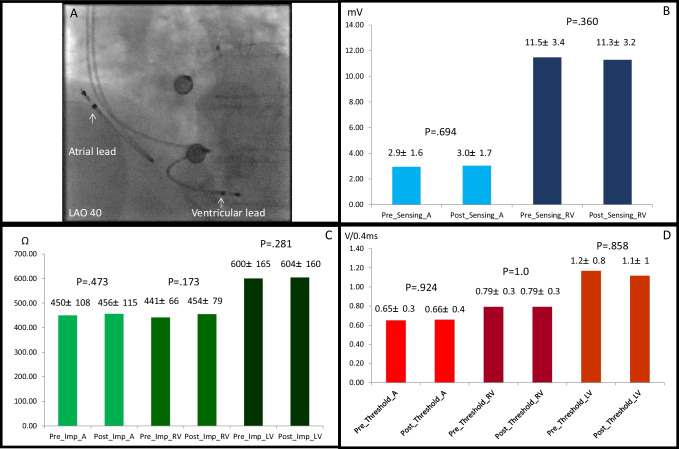
Pre/post-PFA interrogations of the devices

Figure 7A shows no dislodgement of the atrial/ventricular leads after ablation. Figure 7B–D summarize the pre- and post-PFA interrogation of the devices, and there were no significant change of the right atrial sensing 2.9 ± 1.6 mV vs. 3.0 ± 1.7 mV (*P* = 0.694), ventricular sensing 11.5 ± 3.4 mV vs. 11.3 ± 3.2 mV (*P* = 0.360), right atrial impedance 450 ± 108 Ω vs. 456 ± 115 Ω (*P* = 0.473), right ventricular impedance 441 ± 66 Ω vs. 454 ± 79 Ω (*P* = 0.173), left ventricular impedance 600 ± 165Ω vs. 604 ± 160 Ω (*P* = 0.281), right atrial threshold 0.65 ± 0.3 V vs. 0.66 ± 0.4 V (*P* = 0.924), right ventricular threshold 0.79 ± 0.3 V vs. 0.79 ± 0.3 V (*P* = 1.0), and left ventricular threshold 1.2 ± 0.8 V vs. 1.1 ± 1 V (*P* = 0.858).

Table 2 summarizes the procedural findings. The mean diameters of LSPV, LIPV, RIPV, and RSPV were 19.2 ± 3.1 mm, 18.6 ± 3.1 mm, 19.1 ± 3.8 mm, and 18.3 ± 3.2 mm respectively. At the discretion of the operators (mainly based on consideration of the size of the LA or PVs as well as catheter manipulation), 70% of the cases were performed using the 31-mm PFA catheter, and the remaining cases were performed using the 35-mm PFA catheter. The 1.9-kV energy output was initially applied for three patients; thereafter, the recommended 2.0-kV energy output was then applied for the remaining patients. All patients received the “8 applications ablation protocol” (i.e., 4 flowers + 4 baskets), and single-shot isolation was achieved in all PVs. The incidence of bradycardia response was 25% when treating each PV. Notably higher incidence of phrenic nerve capture during ablation was observed at RSPV (90%) and RIPV (90%) than LSPV (20%) and LIPV (10%).

**Table 2:** Summary of procedural findings

LSPV diameter, mm	19.2 ± 3.1
LIPV diameter, mm	18.6 ± 3.1
RIPV diameter, mm	19.1 ± 3.8
RSPV diameter, mm	18.3 ± 3.2
PFA catheter size 31 mm/35 mm, %	70%/30%
PFA energy output 1.9 kV/2.0 kV, %	15%/75%
LSPV applications, *n*	8 (4 in flower pose + 4 in basket pose)
LSPV bradycardia response, %	25%
LSPV phrenic capture, %	20%
LSPV single-shot PVI, %	100%
LIPV applications, *n*	8 (4 in flower pose + 4 in basket pose)
LIPV bradycardia response, %	25%
LIPV phrenic capture, %	10%
LIPV single-shot PVI, %	100%
RIPV applications, *n*	8 (4 in flower pose + 4 in basket pose)
RIPV bradycardia response, %	25%
RIPV phrenic capture, %	90%
RIPV single-shot PVI, %	100%
RSPV applications, *n*	8 (4 in flower pose + 4 in basket pose)
RSPV bradycardia response, %	25%
RSPV phrenic capture, %	90%
RSPV single-shot PVI, %	100%
Procedural time, min	34.9 ± 7.3
Fluoroscopic time, min	7.3 ± 3.1
Fluoroscopy entrance dose-area product, μGym^2^	446.2 ± 221.5

*RSPV*, right superior pulmonary vein; *RIPV*, right inferior pulmonary vein; *LSPV*, left superior pulmonary vein; *LIPV*, left inferior pulmonary vein; *PFA*, pulsed field ablation; *PVI*, pulmonary vein isolation

The mean procedural time (skin-to-skin) was 34.9 ± 7.3 min, fluoroscopic time was 7.3 ± 3.1 min, and mean fluoroscopy entrance dose-area product was 446.2 ± 221.5 μGym^2^. No procedural complications, i.e., death, perforation/tamponade, atrial-esophageal fistula, pulmonary vein stenosis, phrenic nerve injury, stroke, thromboembolic events, myocardial infarction, or major bleeding occurred.

At this stage, only two out of 20 patients had 3-month follow-up, both patients had dual-chamber pacemaker (DDD mode), and the device interrogations (baseline/post-procedural/3 months) showed no significant change of the parameters of the A (atrial lead) and V (ventricular lead) (shown in Table 3). The midterm and long-term follow-up for all patients are under schedule.

**Table 3:** Short-term follow-up for device interrogation from two patients

	Patient 1	Patient 2
Atrial lead sensing		
Baseline	2.3 mV	2 mV
Post-procedural	2.3 mV	2 mV
3 months	2.3 mV	2 mV
Atrial lead impedance		
Baseline	408 Ω	410 Ω
Post-procedural	400 Ω	410 Ω
3 months	400 Ω	410 Ω
Atrial lead threshold		
Baseline	0.75 V 0.4 ms	1 V 0.4 ms
Post-procedural	0.75 V 0.4 ms	1 V 0.4 ms
3 months	0.75 V 0.4 ms	1 V 0.4 ms
Ventricular lead sensing		
Baseline	11.7 mV	11.4 mV
Post-procedural	11.7 mV	11.4 mV
3 months	11.7 mV	11.4 mV
Ventricular lead impedance		
Baseline	550 Ω	490 Ω
Post-procedural	563 Ω	490 Ω
3 months	563 Ω	490 Ω
Ventricular lead threshold		
Baseline	0.75V0.4 ms	1V0.4 ms
Post-procedural	0.75V0.4 ms	1V0.4 ms
3 months	0.75V0.4 ms	1V0.4 ms

## Discussion

### Main findings

**Fig. 8:**
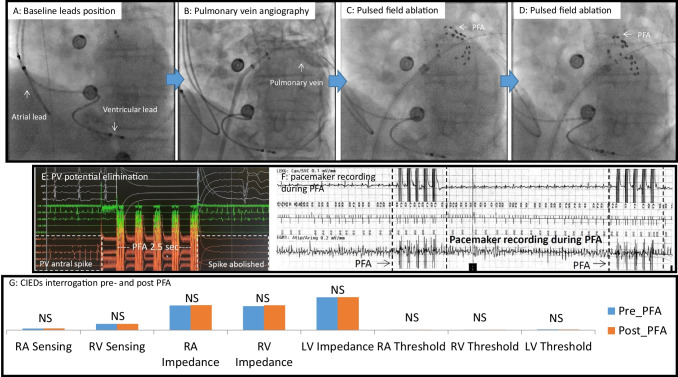
Graphic summary (central illustration)

The key message of this pilot study is summarized in (Graphic Summary) Fig. 8.

To the best of our knowledge, this is the first report of PFA of AF in patients with different CIEDs. Under the guidance of systematic approach, it appeared to be feasible and safe to perform PFA in patients with CIEDs, and the pre- and post-PFA interrogation of the devices showed no significant changes of the parameters/functions of the CIEDs.

### Patient population

AF is the most common arrhythmia in clinical practice, and its prevalence increases significantly with age. Meanwhile, with the aging population, the use of CIEDs has witnessed a steady growth. Catheter ablation has established as an integral component of arrhythmia management [14]. As a result, catheter ablations are becoming more often required in patients with previously implanted CIEDs. The included cohort in our study may represent typical AF patients with implanted CIEDs; the mean age was approximately 72 years old with mean CHA_2_DS_2_-VASc Score of 4.6. All patients were indicated to catheter ablation because of highly symptomatic AF despite AADs therapy.

### To avoid interactions between catheter ablation and CIEDs

Potential interactions between ablation catheters and CIEDs may include (1) alterations in pacing, sensing, or impedance parameters due to energy delivery with direct contact to the leads; (2) oversensing or inappropriate sensing resulting in inappropriate anti-tachycardia pacing and ICD shock; and (3) direct leads dislodgment because of catheter manipulation. Current common understanding is that direct contact between ablation catheters and the CIED systems should be avoided. However, these consensuses are mainly based on precautionary measures, rather than on clinical data. Clinical studies evaluating interactions between CIEDs and ablation catheters remain limited [4]. On the other hand, PFA is a novel, powerful ablation technology using high-voltage electrical field energy, whether PFA has interactions with CIEDs remains unknown.

Prior study of high-voltage electroporation pulses used in other fields such as oncology has been performed in patients with CEIDs without any effect on the device parameters [16]. According to our ablation protocol, PFA should not be performed with direct contact to the CIEDs or other devices. Under this principle, using either 31 mm or 35 mm PFA catheter with output energy ranging from 1.9 kV to 2.0 V, PFA was safely performed in all these patients who had previously implanted different types of devices (pacemaker, ICD, or CRT-D) from different manufactures, indicating general feasibility of PFA in patients with different CIEDs.

### To avoid device dislodgement

As illustrated in the representative figures, before catheter positioning all patients underwent fluoroscopy (under different projections) to identify the baseline position of the atrial/ventricular leads. Guided by a soft, long guiding wire, the long transseptal sheath was advanced and positioned at the superior vena cava without mechanically affecting the position of the leads. Careful transseptal puncture was performed guided by multiple projected fluoroscopies. Interestingly, among the included patients, two patients had previously implanted LAA occluder. Any catheter manipulation which may mechanically dislodge the leads/devices was avoided; and during ablation of all PVs, the PFA catheter was away from the leads and the devices. After the procedure, a fluoroscopy re-examination was performed to confirm no dislodgement of the leads or the devices. For the operators, careful, gentle catheter manipulation should be always kept in mind.

### Procedure safety and efficiency

Consistent with the results from previous studies, the PFA procedure appears to be safe. Due to the anatomic vicinity, we observed significantly higher incidence of phrenic capture of the right-sided PVs (90%) than left-sided PVs (10–20%) during PFA, and no evidence of phrenic injury was detected after the PFA. Since the beginning of the PFA procedure in our center, among the first 52 patients who had post-PFA esophageal endoscopy examination, no patient was found to develop esophageal lesion, after such validation phase, no more esophageal endoscopy was performed after the PFA PVI procedure, and no patients had symptoms suggestive of gastro-esophageal problem during our clinical visit.

Transient bradycardia was not an uncommon phenomenon during the PFA of the PVs, the incidence of transient bradycardia was around one-fourth per PV in this cohort; interestingly, we observed that the bradycardia response occurred relatively seldom among younger patients (e.g., age < 55–60 years).

As a high-volume center, based on our experience, PFA-based PVI represents a simplified and very efficient procedure. Conventionally, in our center, no pre-procedural image was acquired, and we utilize TEE and LA/PV angiography to understand the anatomy. Using the abovementioned “8 applications protocol,” single-shot PVI was achieved at all PVs, and all PVs were confirmed to be electrically isolated during procedural re-mapping. Consistent with our recent reports [17, 18], the PFA-based PVI was performed within a very short procedural time, i.e., the mean skin-to-skin procedural time was only around 35 min, and the procedural fluoroscopic time and dose appeared acceptable, i.e., 7.3 min and 446 μGym2 respectively. Nonetheless, such extreme fast procedure time may also be partly explained by the proficiency of our primary operators who all had > 3000 ablations experience with different ablation technologies.

## Limitations

This was not a multicenter randomized trial although all the indicated patients were consecutively included without subjective selection bias. As a pilot proof-of-concept study, the sample size was small indeed. The present study only referred to the FARAPULSE Technology; thus, the results may not be generalized to other PFA technologies. The present study mainly focused on the practical approach, procedural feasibility and safety, and long-term clinical outcome remains under investigation. PFA was only applied for PVI in this cohort; therefore, the results of this technical report may not be extrapolated to non-PVI application.

## Conclusions

This pilot cohort study for the first time reports the PFA-based PVI in AF patients with different CIEDs. Under the guidance of systematic approach, it appears to be feasible and safe to perform PFA in patients with CIEDs, and the pre- and post-PFA interrogation of the devices showed no significant alterations of the parameters/functions of the CIEDs. Our data may provide initial evidence for further studies of PFA in patients with CIEDs.

## Data Availability

Data are available on reasonable request to the corresponding author.

## References

[1] Hindricks G, Potpara T, Dagres N, Arbelo E, Bax JJ, Blomström-Lundqvist C, Boriani G, Castella M, Dan GA, Dilaveris PE, Fauchier L, Filippatos G, Kalman JM, La Meir M, Lane DA, Lebeau JP, Lettino M, Lip GYH, Pinto FJ, Thomas GN, Valgimigli M, Van Gelder IC, Van Putte BP, Watkins CL; ESC Scientific Document Group. 2020 ESC Guidelines for the diagnosis and management of atrial fibrillation developed in collaboration with the European Association for Cardio-Thoracic Surgery (EACTS): The Task Force for the diagnosis and management of atrial fibrillation of the European Society of Cardiology (ESC) Developed with the special contribution of the European Heart Rhythm Association (EHRA) of the ESC. Eur Heart J. 2021 Feb 1;42(5):373–498. 10.1093/eurheartj/ehaa612. Erratum in: Eur Heart J. 2021 Feb 1;42(5):507. Erratum in: Eur Heart J. 2021 Feb 1;42(5):546–547. Erratum in: Eur Heart J. 2021 Oct 21;42(40):4194. 10.1093/eurheartj/ehab648 34520521

[2] Chen S, Pürerfellner H, Meyer C, Acou WJ, Schratter A, Ling Z, Liu S, Yin Y, Martinek M, Kiuchi MG, Schmidt B, Chun KRJ (2020). Rhythm control for patients with atrial fibrillation complicated with heart failure in the contemporary era of catheter ablation: a stratified pooled analysis of randomized data. Eur Heart J.

[3] Chen S, Pürerfellner H, Ouyang F, Kiuchi MG, Meyer C, Martinek M, Futyma P, Zhu L, Schratter A, Wang J, Acou WJ, Ling Z, Yin Y, Liu S, Sommer P, Schmidt B, Chun JKR (2021). Catheter ablation vs antiarrhythmic drugs as ‘first-line’ initial therapy for atrial fibrillation: a pooled analysis of randomized data. Europace.

[4] Darrat YH, Morales GX, Elayi CS (2017). The effects of catheter ablation on permanent pacemakers and implantable cardiac defibrillators. J Innov Card Rhythm Manag..

[5] Shaojie Chen Yuehui, Zhiyu Yin, Christian Ling, Helmut Meyer, Martin Pürerfellner, Galindo Martinek Márcio, Piotr Kiuchi, Lin Futyma, Alexandra Zhu, Jiazhi Schratter, Willem-Jan Wang, Philipp Acou, Feifan Sommer, Shaowen Ouyang, Liu Julian KR, Chun Boris, Schmidt. Evolving role of catheter ablation for atrial fibrillation: early and effective rhythm control. J Clin Med. 2022;11(22):6871. 10.3390/jcm11226871. 10.3390/jcm11226871 PMC9696051 36431348

[6] Verma A, Jiang CY, Betts TR, Chen J, Deisenhofer I, Mantovan R, Macle L, Morillo CA, Haverkamp W, Weerasooriya R, Albenque JP, Nardi S, Menardi E, Novak P, Sanders P (2015). STAR AF II Investigators Approaches to catheter ablation for persistent atrial fibrillation. N Engl J Med.

[7] Chen S, Schmidt B, Bordignon S, Tohoku S, Urban VC, Schulte-Hahn B, Chun KRJ (2021). Catheter ablation of atrial fibrillation using ablation index-guided high-power technique: Frankfurt AI high-power 15-month follow-up. J Cardiovasc Electrophysiol.

[8] Cochet H, Nakatani Y, Sridi-Cheniti S, Cheniti G, Ramirez FD, Nakashima T, Eggert C, Schneider C, Viswanathan R, Derval N, Duchateau J, Pambrun T, Chauvel R, Reddy VY, Montaudon M, Laurent F, Sacher F, Hocini M, Haïssaguerre M, Jais P (2021). Pulsed field ablation selectively spares the oesophagus during pulmonary vein isolation for atrial fibrillation. Europace.

[9] Koruth J, Kuroki K, Iwasawa J, Enomoto Y, Viswanathan R, Brose R, Buck ED, Speltz M, Dukkipati SR, Reddy VY (2019). Preclinical evaluation of pulsed field ablation: electrophysiological and histological assessment of thoracic vein isolation. Circ Arrhythm Electrophysiol.

[10] Koruth JS, Kuroki K, Kawamura I, Brose R, Viswanathan R, Buck ED, Donskoy E, Neuzil P, Dukkipati SR, Reddy VY (2020). Pulsed field ablation versus radiofrequency ablation: esophageal injury in a novel porcine model. Circ Arrhythm Electrophysiol.

[11] Reddy VY, Koruth J, Jais P, Petru J, Timko F, Skalsky I, Hebeler R, Labrousse L, Barandon L, Kralovec S, Funosako M, Mannuva BB, Sediva L, Neuzil P (2018). Ablation of atrial fibrillation with pulsed electric fields: an ultra-rapid, tissue-selective modality for cardiac ablation. JACC Clin Electrophysiol.

[12] Reddy VY, Neuzil P, Koruth JS, Petru J, Funosako M, Cochet H, Sediva L, Chovanec M, Dukkipati SR, Jais P (2019). Pulsed field ablation for pulmonary vein isolation in atrial fibrillation. J Am Coll Cardiol.

[13] Reddy VY, Dukkipati SR, Neuzil P, Anic A, Petru J, Funasako M, Cochet H, Minami K, Breskovic T, Sikiric I, Sediva L, Chovanec M, Koruth J, Jais P (2021). Pulsed field ablation of paroxysmal atrial fibrillation: 1-year outcomes of IMPULSE, PEFCAT, and PEFCAT II. JACC Clin Electrophysiol.

[14] Schmidt B, Chen S, Tohoku S, Bordignon S, Bologna F, Chun KRJ. Single shot electroporation of premature ventricular contractions from the right ventricular outflow tract. Europace. 2021 Sep 18:euab212. 10.1093/europace/euab212. 10.1093/europace/euab212 34536008

[15] Tohoku S, Schmidt B, Bordignon S, Chen S, Bologna F, Urbanek L, Pansera F, Chun KRJ (2022). Pulsed field ablation for persistent superior vena cava: new solution for an old problem. JACC Case Rep.

[16] Jarm T, Krmac T, Magjarevic R, Kos B, Cindric H, Miklavcic D (2020). Investigation of safety for electrochemotherapy and irreversible electroporation ablation therapies in patients with cardiac pacemakers. Biomed Eng Online.

[17] Chen S, Schmidt B, Bordignon S, Tohoku S, Urbanek L, Schaack D, Chun JKR. (2022) Pulsed field ablation as first-line “efficient” rhythm control for atrial fibrillation complicated with heart failure: proof-of-concept. J Interv Card Electrophysiol. 10.1007/s10840-022-01398-4 10.1007/s10840-022-01398-4 36241936

[18] Shaojie, Chen Boris, Schmidt Stefano, Bordignon Shota, Tohoku Lukas, Urbanek Julian K. R., Chun Pulsed field ablation as first‐line treatment to reduce atrial fibrillation burden documented by pacemaker. Pacing and Clinical Electrophysiology Pace. 14629. 10.1111/pace.14629 10.1111/pace.14629 36424836

